# A Randomized, Endoscopist-Blinded, Prospective Trial to Compare the Efficacy and Patient Tolerability between Bowel Preparation Protocols Using Sodium Picosulfate Magnesium Citrate and Polyethylene-Glycol (1 L and 2 L) for Colonoscopy

**DOI:** 10.1155/2020/9548171

**Published:** 2020-03-03

**Authors:** Sang Hoon Kim, Ji Hyeong Kim, Bora Keum, Han Jo Jeon, Se Hyun Jang, Seong Ji Choi, Seung Han Kim, Jae Min Lee, Hyuk Soon Choi, Eun Sun Kim, Yoon Tae Jeen, Hong Sik Lee, Hoon Jai Chun, Chang Duck Kim

**Affiliations:** Division of Gastroenterology and Hepatology, Department of Internal Medicine, Korea University College of Medicine, Anam-dong, Seongbuk-gu, Seoul 20841, Republic of Korea

## Abstract

Patient compliance during bowel preparation is important for successful colonoscopy. Bowel preparation with polyethylene glycol (PEG), the most commonly used solution for cleansing, involves the unpleasant ingestion of a large amount of liquid. Sodium picosulfate magnesium citrate (SP-MC) solution is an alternative option with better palatability than PEG. Therefore, in this study, we compared the efficacy and patient tolerability among the following three bowel preparation protocols: 2 L PEG-ascorbic acid (ASc), 1 L PEG-ASc plus bisacodyl, and SP-MC 340 mL plus bisacodyl. We conducted a randomized prospective endoscopist-blinded study between August 2018 and January 2019. A total of 311 patients were randomly classified into three groups according to the above-described bowel preparation protocols. To evaluate the efficacy of bowel cleansing, we used the Boston Bowel Preparation Scale. The degree of symptoms and the patients' satisfaction with each bowel preparation method were investigated using a questionnaire completed before sedation for colonoscopy. The baseline characteristics were similar among the three groups. There was no significant difference in the bowel preparation quality among the three groups. However, the incidence of symptoms, such as abdominal fullness and pain, was significantly lower (*P* = 0.006 and 0.027, respectively) while the patients' satisfaction rate was significantly higher (*P* = 0.012) in the SP-MC plus bisacodyl group than in the two PEG groups. In this study, the efficacy of the SP-MC plus bisacodyl solution was similar to that of the PEG solutions. However, patient tolerability and satisfaction were better in the SP-MC plus bisacodyl group than in the other groups. In conclusion, the use of SP-MC plus bisacodyl bowel preparation solution might be a better method for providing good intestinal cleansing and improving patient compliance.

## 1. Introduction

Detection and treatment of polyps through periodic colonoscopy play a major role in reducing the incidence of malignant colorectal cancer [1]. Many studies [2–5] have reported that poor bowel preparation can lead to an increased number of missed adenomas, incomplete procedures, and prolonged cecal intubation time. However, patients are often reluctant to undergo colonoscopy because of the unpleasant experiences during bowel cleansing.

Ideally, bowel cleansing should be able to empty the colon without affecting the mucosa and cause minimal discomfort while avoiding electrolyte imbalance [6, 7]. Until recently, the 4 L polyethylene glycol- (PEG-) based bowel preparation method was widely used since its introduction in 1980 [8]. However, the use of large amounts of PEG, which has poor palatability, was a major factor that decreased the patients' compliance with the preparation process and their satisfaction with the procedure. An alternative strategy for reducing the volume to 2 L has been proposed, and many studies have reported that 2 L PEG in split doses is not inferior to 4 L PEG in terms of efficacy [9, 10]. On the basis of these results, the use of low-dose purgatives, such as 2 L PEG, can also be recommended as an alternative to the standard 4 L split-dose regimen during screening colonoscopy for healthy individuals [11].

Despite efforts to improve compliance with the PEG-based solutions, ingestion of PEG-ascorbic acid (PEG-ASc) is still a burden to many patients, especially old patients, those showing poor performance and those with comorbidities. Our team has previously conducted a comparison study between the 2 L PEG-ASc and 1 L PEG-ASc plus bisacodyl methods [12] and reported that the efficacy was similar between the two groups. An adjunctive agent, simethicone, has also been reported to further improve the efficacy of cleansing while using low-volume PEG in a multicenter clinical trial [13]. Simultaneously, efforts to find new and better drugs for bowel preparation are ongoing. Sodium picosulfate magnesium citrate (SP-MC), a low-volume bowel cleanser, was approved by the United States Food and Drug Administration in July 2012. Sodium picosulfate induces hyperperistalsis, whereas hyperosmotic magnesium citrate increases the luminal water volume [14]. SP-MC was generally better accepted than the split-dose PEG regimen in many prospective randomized studies [15, 16]. However, a study suggested that the efficacy of SP-MC alone was relatively inferior to that of PEG-ASc [17]. Therefore, we used bisacodyl as an adjunct to SP-MC to facilitate bowel cleansing in this study. Bisacodyl is an unabsorbable diphenylmethane derivative with stimulant laxative properties [18]. We hypothesized that the SP-MC plus bisacodyl method may decrease, both the dosage of the laxative solution (340 mL) and the total volume that must be ingested compared with the 2 L PEG-ASc method (2.34 L vs. 3 L). As described above, there is currently no gold standard for bowel cleansing. In this study, we set the 2 L PEG-ASc method as the control group (group A) and compared the patients' compliance and the efficacy of bowel preparation with the 1 L PEG-ASc plus bisacodyl method (group B) and SP-MC plus bisacodyl method (group C).

## 2. Materials and Methods

### 2.1. Study Design

We conducted a single-center prospective endoscopist-blinded randomized study at the Department of Gastroenterology and Digestive Endoscopy, Korea University Anam Hospital (Seoul, Korea), from August 2018 to January 2019. We prospectively enrolled patients and randomly assigned them into the three groups (1 : 1 : 1 ratio) using a computer-generated designation system. All patients were given written instructions and guidelines, and they all provided written informed consent. This trial was approved by the institutional review board of the Korea University Anam Hospital Clinical Trial Center (#2018AN0235). The study was registered as a clinical trial on August 20, 2019 (#KCT0004218, Korean Clinical Trials Registry at the Korea Centers for Disease Control and Prevention).

### 2.2. Patients

**Figure 1:**
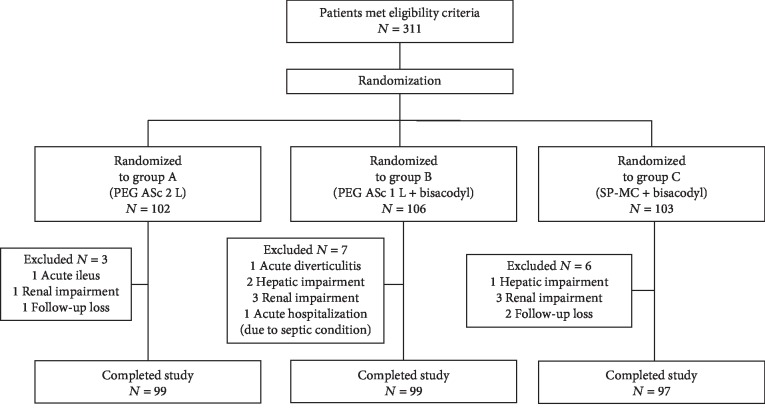
Flow diagram of patient enrollment.

This study consecutively enrolled 311 patients aged 18–74 years who had different indications for colonoscopy. As there was no definite evidence for the stability and safety of SP-MC for the elderly population, we limited the patients' age to 74 years. Considering the risk of electrolyte imbalance and acute hepatitis, patients with a history of renal or hepatic dysfunction were excluded from the study. Additionally, patients were excluded if they had a history of a gastrointestinal obstruction, ileus, severe heart failure, uncontrolled hypertension (systolic blood pressure > 170 mmHg, diastolic blood pressure > 100 mmHg), prior bowel resection, or gastroparesis. The participants were enrolled by a study coordinator. Computer-generated randomization was used to ensure equal distribution into the three different types of bowel preparation. Laboratory tests, including determination of creatinine, electrolyte, and alanine aminotransferase levels, were performed before the cleansing process. Patients were later excluded from the study according to the precleansing laboratory results (serum creatinine ≥ 2.0 mg/dL and alanine aminotransferase > 80 IU/L). Therefore, a total of 295 patients completed the study (Figure 1). The endoscopists and investigators were blinded to the allocation groups.

### 2.3. Colonoscopy Preparation and Diet Protocol

**Figure 2:**
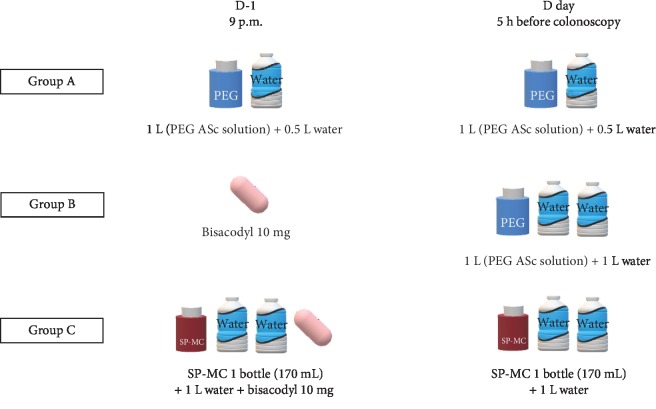
Methods for bowel preparation.

All patients in all three groups received the same instructions on how to prepare for the test, restrict the diet, and ingest the solution for bowel preparation. The participants were not allowed to take indigestible fiber-rich food (such as vegetables and fruits) for 3 days before the scheduled colonoscopy. A light dinner on the day before the procedure was allowed. Total colonoscopy was performed between 9 a.m. and 5 p.m. on the scheduled date of the test. The three protocols of bowel preparation are described in Figure 2.

In group A (2 L PEG in split doses), the patients were instructed to ingest 1 L of PEG solution (Coolprep; Taejoon Pharm. Inc., Seoul, Korea; containing 2.691 g sodium chloride, 1.015 g potassium chloride, 7.5 g sodium sulfate, 100 g PEG, 4.7 g ascorbic acid, and 5.9 g/L sodium ascorbate) at 9 p.m. on the day before the procedure with 0.5 L of water. The patients ingested the remaining 1 L of PEG and 0.5 L of water 5 h before the colonoscopy procedure.

In group B (1 L PEG with bisacodyl), the patients took 10 mg bisacodyl (Dulcolax; Sanofi-Aventis Korea Inc., Seoul, Korea) at 9 p.m. on the day before the procedure. The patients ingested 1 L of PEG and 1 L of water 5 h before the colonoscopy procedure.

In group C (SP-MC with bisacodyl), the patients ingested one bottle of 170 mL SP-MC (Picosolution; Pharmbio Korea Co., Ltd., Seoul, Korea) with 1 L of water and 10 mg bisacodyl at 9 p.m. on the day before the procedure. They ingested another bottle of SP-MC with 1 L of water 5 h before the colonoscopy procedure.

The investigators carefully instructed all patients to take >90% of the total dose of laxatives and to follow the exact instructed timing of dosing.

### 2.4. Assessment by Endoscopists: Efficacy of Bowel Cleansing

Colonoscopy was performed by three expert endoscopists who were blinded to the preparation regimen. These three endoscopists had worked for >5 years at our hospital and had performed >1000 colonoscopy procedures per year, including therapeutic procedures, such as colonic endoscopic submucosal dissection.

After colonoscopy, each endoscopist evaluated the degree of bowel cleansing according to the Boston Bowel Preparation Scale (BBPS). The BBPS is a four-point scoring system that applies to each of the three segments of the colon [9, 10, 12, 14], as follows: 0—unprepared colon segment with the mucosa not visible because of solid stool that could not be cleared; 1—a portion of the mucosa of the colon segment is visible, but other areas of the colon segment are not clearly visible because of staining, residual stool, and/or opaque liquid; 2—minor amount of residual staining, small fragments of stool, and/or opaque liquid, but the mucosa of the colon segment is clearly visible; and 3—the entire mucosa of the colon segment is clearly visible, with no residual staining, small fragments of stool, or opaque liquid. The BBPS score was recorded before irrigation or suction, thus providing a direct evaluation of the effectiveness of the bowel preparation. The bowel preparation status was compared using the mean score from all the groups.

### 2.5. Assessment of Patient Tolerability and Adverse Events

The patients' tolerability and adverse events were assessed using a brief questionnaire completed before the endoscopic examination to evaluate the patients' comfort and compliance with respect to the bowel preparation. The patients were asked to complete the provided questionnaire. We evaluated the patients for abdominal discomfort, abdominal pain, nausea, vomiting, and sleep disturbance. The degree of discomfort was assessed on a five-point numerical scale (1, none; 2, mild; 3, moderate; 4, severe; and 5, very severe). The satisfaction score for each preparation protocol was derived using the percentage of patients who felt that the method was more comfortable than their previous colonoscopy experience. Patients with no history of previous colonoscopy were excluded from the survey.

### 2.6. Sample Size and Statistical Analysis

The adequate sample size required for the study was estimated on the basis of the results of a separate pilot study that included 15 patients per group. According to the pilot study, the bowel preparation quality recorded using the BBPS score for each group were as follows: 7.33 for group A, 7.60 for group B, and 6.07 for group C. Considering these results, a sample size of at least 91 patients was required for each treatment group to detect a difference in treatment effect with a 5% type I error rate and 80% power. We decided to enroll at least 100 patients per treatment group considering a 10% dropout rate in the study. We used G^∗^Power version 3.1.9.2 for sample size calculation.

In this study, the data are presented as mean values and standard deviations for continuous variables or as counts and percentages for discontinuous variables. In the analysis for the three different groups, one-way analysis of variance was used to compare the continuous variables, and either chi-square statistics or Fisher's exact tests were used for categorical data. We used SPSS version 20.0 for data entry and statistical analysis. A *P* value < 0.05 was considered statistically significant.

## 3. Results

### 3.1. Baseline Characteristics

A total of 295 patients who were assigned into the three groups completed this study. The baseline characteristics of age, height, weight, body mass index, baseline medical conditions, and indication for colonoscopy were similar among the groups (Table 1). The average time interval between the latest colonoscopy procedures was 3.88 years, and there was no statistical difference among the three groups. Furthermore, there were no significant differences among the three groups in terms of strict food restriction, fasting time, cecal intubation success rate, total examination time, and adenomatous polyp detection rate (Table 2).

### 3.2. Efficacy of Bowel Cleansing and Interobserver Variation

**Figure 3:**
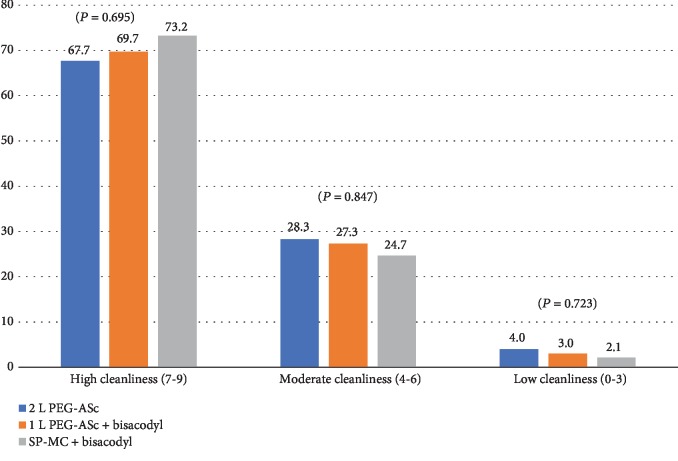
Efficacy of bowel preparation. BBPS distribution is expressed in %. BBPS: Boston Bowel Preparation Scale.

**Figure 4:**
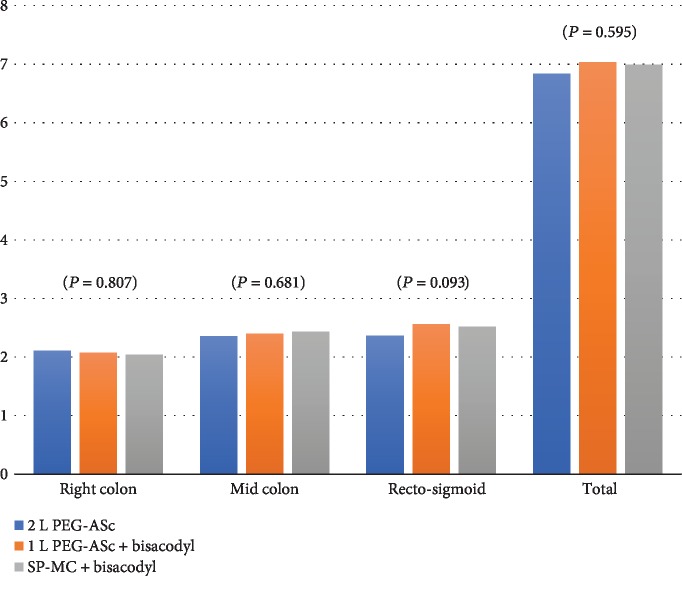
Boston Bowel Preparation Scale (BBPS) scores for each colon segment.

A total BBPS score ≥ 6 with all colon segments scoring > 2 was considered “adequate” bowel preparation [19, 20]. There was no significant difference in the proportion of patients who achieved a score of ≥6 in each group (*P* = 0.917; Table 3, Figure 3). The comparison of the segmental BBPS scores (right colon, middle colon, and rectosigmoid) showed similar findings in all groups for the ascending colon and cecum (2.11 ± 0.768 vs. 2.07 ± 0.811 vs. 2.04 ± 0.660, *P* = 0.807), transverse colon (2.35 ± 0.577 vs. 2.40 ± 0.699 vs. 2.43 ± 0.644, *P* = 0.681), and rectosigmoid (2.36 ± 0.646 vs. 2.56 ± 0.688 vs. 2.52 ± 0.614, *P* = 0.093) (Table 3, Figure 4). Consistently, there was no significant difference in the sum of the scores for the three segments. There was no significant difference in the BBPS score among the three endoscopists (*P* = 0.400). The proportion of endoscopists who performed colonoscopy did not show a difference among the three preparation groups.

### 3.3. Patients' Tolerability and Adverse Events

**Figure 5:**
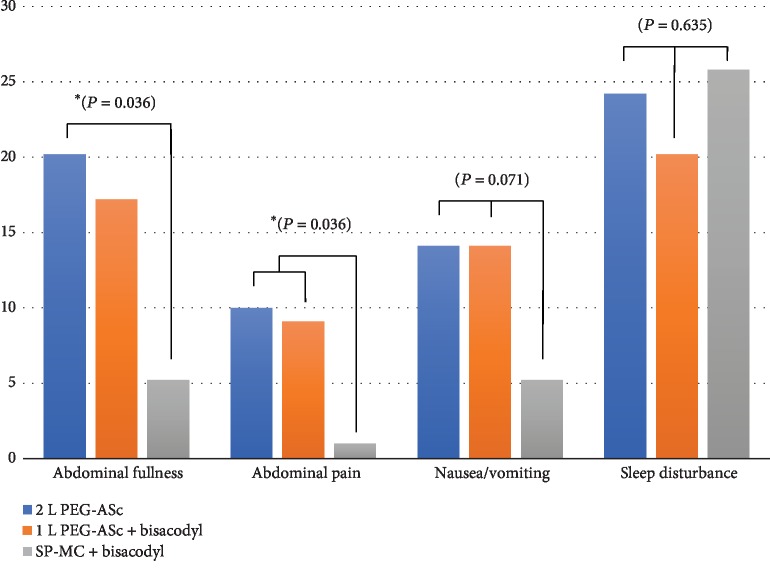
Patient tolerability and reports of adverse effects (%).

**Figure 6:**
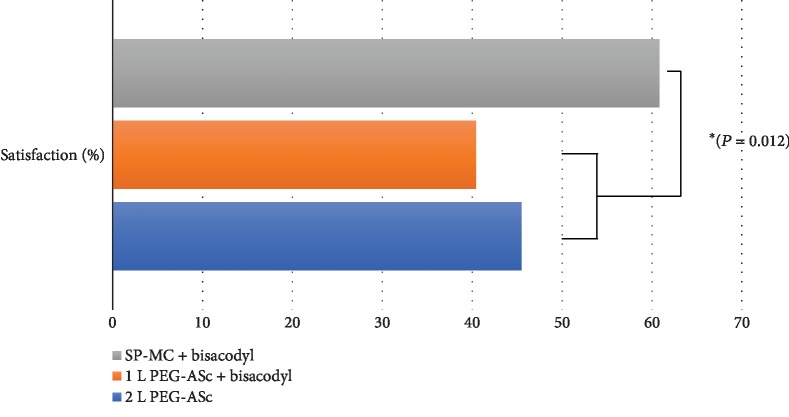
Patient satisfaction score.

We compared the patients' discomfort during the preparation for colonoscopy among the groups. We compared the relative proportion of patients complaining of symptoms of moderate (numerical scale 3) or greater severity for each group using Pearson's chi-square test. There were statistical differences in the severity of the patients' symptoms, such as abdominal fullness (*P* = 0.006) and abdominal pain (*P* = 0.027) among the three groups (Figure 5). To identify the group that contributed to this difference, post hoc testing using adjusted *z*-scores was performed. The results revealed that between the 2 L PEG group and the SP-MC plus bisacodyl group, there was a significant difference in the proportion of patients who experienced moderate to severe abdominal fullness (*P* = 0.036). In terms of abdominal pain, the SP-MC group showed a statistically significant difference from both PEG-based groups. Although nausea or vomiting tended to occur less frequently in the SP-MC group, no significant intergroup differences were noted (*P* = 0.071). Furthermore, we compared the patients' satisfaction with the current colonoscopy preparation with that for a previous colonoscopy preparation. Of the total 295 patients, 22 underwent colonoscopy for the first time; thus, this comparison was performed with the remaining 273 patients who had a previous colonoscopy experience. The results showed that the satisfaction rate was significantly higher in the SP-MC group than in the other groups (40.18 ± 0.485% vs. 45.53 ± 0.488% vs. 60.57 ± 0.493%, *P* = 0.012; Figure 6). As the bowel cleansing efficacy and the patients' symptoms during preparation may vary depending on the presence and type of the final gastrointestinal disease, we reviewed the distribution of the final diagnosis made according to the results of endoscopy, pathology, and computed tomography.  Table 4 shows the distribution; no significant intergroup difference was observed.

## 4. Discussion

In this study, there was no significant difference in the quality of bowel cleansing, as assessed using the BBPS score, among the three groups. It was noteworthy that the SP-MC solution with 10 mg bisacodyl was not statistically inferior to the 2 L PEG-ASc solution in terms of mucosal visualization. The SP-MC group showed significantly less abdominal discomfort and pain than did the other two groups using PEG-ASc, whereas there was no significant difference in other types of discomfort. Although the 1 L PEG-ASc group had the least total volume of preparation solution (2 L), the abdominal symptoms were more frequent in this group than in the SP-MC group (which had a total of 2.34 L preparation solution). According to our data, the SP-MC solution seemed to have achieved higher patient satisfaction and compliance owing to the combined effect of its high palatability and smaller volume. Although the three groups showed similar bowel preparation quality, patients who used the SP-MC with bisacodyl preparation solution reported higher satisfaction with the current cleansing method for colonoscopy than with their previous colonoscopy experience.

We additionally investigated the factors that might be involved in insufficient bowel preparation in our study population. As the study was not designed for logistic regression analysis, we analyzed the characteristics of patients with insufficient bowel preparation in a retrospective manner. Compared with the controls, the patients with insufficient bowel preparation showed a tendency toward a higher score for nausea/vomiting (*P* = 0.053) and abdominal pain (*P* = 0.007), which could have led to poor compliance with the use of cleansing solutions. Therefore, our data suggest that it is possible to obtain an acceptable BBPS score (i.e., noninferior to PEG-based protocols) without taking the poorly palatable PEG-ASc solution by improving compliance with bowel preparation using a relatively small amount of a laxative solution (SP-MC, 340 mL total, 170 mL per each dose).

However, in the clinical setting, several potential adverse effects related to the intake of the SP-MC solution should be considered before its prescription. Many reports have suggested that sodium picosulfate use is associated with an increased risk of hyponatremia in older adults [21, 22]. However, Seinelä et al. [23] found no differences in their comparative analysis of the efficacy and safety profiles of the SP-MC and PEG solutions in elderly patients. Owing to the existing controversy, SP-MC is used for elderly patients with caution. In addition, PEG is preferred over SP-MC in individuals with impaired renal function or patients with heart failure. The higher risk of renal dysfunction deterioration and electrolyte imbalance in patients with preexisting renal dysfunction due to sodium picosulfate use than due to PEG use is well-known [24]. As SP-MC may also lead to aggravation of heart failure via absorption [25], careful observation is also required in patients with decreased ejection fraction.

There are some limitations of this study. First, there was no postprocedural laboratory test for the confirmation of any electrolyte or osmolarity imbalance that commonly accompanies the first enema procedure. However, patients who had underlying diseases that could lead to electrolyte abnormalities, such as chronic kidney disease and liver cirrhosis, were excluded from the study; therefore, it was estimated that the possibility of an electrolyte abnormality during bowel cleansing was low. Considering that the chart reviews showed that there was no patient hospitalized within 30 days after colonoscopy with serious hyponatremia or change in mental status, we believe that a meaningful electrolyte imbalance due to bowel cleansing did not occur. Second, as this was a single-center study including Korean patients only, our results cannot be widely applied to patients of different ethnicities and socioeconomic status. Given that this was a single-center study showing a relatively small difference in the cleansing efficacy among the three groups, the verification power might have been negatively affected by the sample size. In a recent multicenter clinical trial, the efficacy of 2 L PEG cleansing was inferior to that of 3 L split preparation in a Chinese population [26]. In this regard, multicenter studies with different patient groups should be performed to improve the representativeness of the sample population. In addition, some factors that could affect bowel cleansing were not analyzed. For example, medication history, including the use of tricyclic antidepressants or narcotics, is known to affect the bowel preparation quality. These unknown factors may have differently affected each group. Third, the study involved a relatively simple evaluation of patient satisfaction by comparing with a previous colonoscopy experience. Considering that colonoscopy is performed every 5 years for the general population in Korea, most of the patients have had an experience where the 2 L PEG (or less frequently 4 L PEG) solution was used during a previous colonoscopy. It was highly unlikely that any of the patients had previously used the 1 L PEG-ASc plus bisacodyl or the SP-MC plus bisacodyl solution. Therefore, we believe that the satisfaction with a previous endoscopy was sufficient to suggest the patients' satisfaction with the new method. However, the inability to accurately confirm the previous method of bowel cleansing was a limiting factor in estimating the relative satisfaction with the bowel preparation. Furthermore, we also believe that a more meaningful analysis would be possible if each type of discomfort experienced during this procedure was compared with the discomfort experienced during the previous colonoscopy. However, not all patients had relevant records because some patients underwent endoscopy elsewhere, and not all patients could remember the name and amount of the previously used bowel preparation solution. In a follow-up study, we plan to devise ways to confirm the patients' past bowel preparation methods to allow a more meaningful comparative analysis. Additionally, as BBPS is a validated tool for postwash scoring, it would have been better to use a prewash scoring system, such as the Aronchick Bowel Preparation Scale, along with the BBPS [27–30].

## 5. Conclusion

In conclusion, the combination of SP-MC plus bisacodyl has a similar bowel cleansing ability as the 1 and 2 L PEG-ASc solutions. The results of this study suggest that, for patients without contraindications, bowel preparation using SP-MC plus bisacodyl can lead to a BBPS score similar to that achieved using the PEG solutions and can improve the patients' compliance by reducing discomfort.

## Figures and Tables

**Table 1:** Baseline characteristics.

	Group A (*N* = 99)	Group B (*N* = 99)	Group C (*N* = 97)	*P* value
Strict food restriction (no. (%))	25 (25.3%)	29 (29.3%)	22 (22.7%)	0.565
Age (mean ± SD (years))	58.09 ± 13.15	54.35 ± 12.92	56.51 ± 1.41	0.140
Height (mean ± SD (m))	1.65 ± 0.09	1.64 ± 0.09	1.66 ± 0.08	0.209
Weight (mean ± SD (kg))	63.35 ± 11.0	63.86 ± 10.9	63.47 ± 9.8	0.941
BMI (mean ± SD (kg))	23.14 ± 3.19	23.58 ± 3.37	22.88 ± 2.62	0.277
Comorbidities (no. (%))				
Hypertension	31 (31.3%)	28 (28.3%)	32 (33.0%)	0.770
Diabetes mellitus	13 (13.1%)	12 (12.1%)	17 (17.5%)	0.517
Cardiovascular disease	10 (10.1%)	11 (11.1%)	8 (8.2%)	0.792
Others	33 (33.3%)	29 (29.3%)	25 (25.8%)	0.509
Indication for colonoscopy				
Screening/surveillance	55 (56.1%)	50 (50.5%)	60 (61.9%)	0.278
History of colon polyp	22 (22.2%)	24 (24.2%)	20 (20.6%)	0.830
Abdominal pain	5 (5.1%)	10 (10.1%)	4 (4.1%)	0.184
Overt intestinal bleeding	1 (1.0%)	1 (1.0%)	2 (2.1%)	0.764
Occult blood in stool	1 (1.0%)	2 (2.0%)	1 (1.0%)	0.782
Loose stool	4 (4.0%)	2 (2.0%)	6 (6.2%)	0.336
Anemia	2 (2.0%)	2 (2.0%)	2 (2.1%)	1.000
Others	9 (9.0%)	8 (8.0%)	5 (5.2%)	0.855
Constipation^∗^	14 (14.1%)	16 (16.2%)	16 (16.3%)	0.895
Education (more than high school education)	69 (69.7%)	59 (59.6%)	64 (65.3%)	0.328
Currently single (unmarried/divorced/bereaved)	28 (28.3%)	33 (33.3%)	27 (27.6%)	0.626
Time interval from previous colonoscopy (years)	4.20	3.76	3.70	0.845

Group A: 2 L PEG-ASc; Group B: 1 L PEG-ASc plus bisacodyl; Group C: SP-MC plus bisacodyl; SD: standard deviation; BMI: body mass index. ^∗^Constipation was defined as a defecation frequency of less than once every 3 days.

**Table 2:** Results of indicators related to colonoscopy.

	Group A (*N* = 99)	Group B (*N* = 99)	Group C (*N* = 97)	*P* value
Strict food restriction (no. (%))	25 (25%)	29 (29.3%)	22 (22.7%)	0.427
NPO time (mean ± SD (h))	16.89 ± 3.84	16.09 ± 2.68	16.98 ± 3.48	0.127
Interval time between the last dose of solution and the procedure (h)	6.21	6.11	6.13	0.763
Cecal intubation success (%)	100	100	100	1
Cecal intubation time (mean ± SD (min))	6.99 ± 2.95	6.35 ± 2.00	6.72 ± 2.23	0.182
Total examination time (mean ± SD (min))	24.31 ± 7.98	22.07 ± 9.55	23.84 ± 6.89	0.132

Group A: 2 L PEG-ASc; Group B: 1 L PEG-ASc plus bisacodyl; Group C: SP-MC plus bisacodyl; SD: standard deviation; NPO: not per oral.

**Table 3:** The Boston Bowel Preparation Scale scores for each patient group.

	Group A (*N* = 99)	Group B (*N* = 99)	Group C (*N* = 97)	*P* value
Ascending colon and cecum	2.11 ± 0.768	2.07 ± 0.811	2.04 ± 0.660	0.807
Transverse colon	2.35 ± 0.577	2.40 ± 0.699	2.43 ± 0.644	0.681
Rectosigmoid	2.36 ± 0.646	2.56 ± 0.688	2.52 ± 0.614	0.093
Total BBPS score	6.84 ± 1.390	7.03 ± 1.508	6.99 ± 1.271	0.595
Adequate bowel preparation (no. (%))	85 (85.9%)	85 (85.9%)	85 (87.6%)	0.917

Group A: 2 L PEG-ASc; Group B: 1 L PEG-ASc plus bisacodyl; Group C: SP-MC plus bisacodyl; BBPS: Boston Bowel Preparation Scale.

**Table 4:** Comparison between groups: final diagnosis of patients undergone colonoscopy.

Final diagnosis	Preparation			*P* value
	Group A	Group B	Group C	
1. Normal	44 (44.4%)	35 (35.4%)	46 (47.4%)	0.203
2. Colon adenoma/cancer	45 (45.5%)	48 (48.5%)	36 (37.1%)	0.252
3. Irritable bowel syndrome	13 (11.0%)	18 (16.8%)	11 (9.9%)	0.351
4. Colonic diverticula	9 (7.6%)	7 (6.5%)	4 (3.6%)	0.371
5. GI hemorrhage	4 (3.4%)	6 (5.6%)	1 (0.9%)	0.170
6. Hemorrhoids	6 (5.1%)	2 (1.9%)	5 (4.5%)	0.350
7. Colitis	6 (5.1%)	4 (3.7%)	5 (4.5%)	0.810
8. Newly diagnosed IBD (UC/CD)	1 (0.8%)	1 (0.9%)	3 (2.7%)	0.436
9. Known IBD (UC/CD)	0 (0.0%)	1 (0.9%)	3 (2.7%)	0.166
10. Others	5 (4.2%)	4 (3.7%)	6 (5.4%)	0.801

1. Normal: includes small hyperplastic polyps approved by pathology; 10. Others: includes gastrointestinal tract lymphoma, melanosis coli, cholecystitis and pancreatic neoplasm, gastric adenoma, or malignancy. Multiple diagnoses per person were possible. GI: gastrointestinal; IBD: inflammatory bowel disease; UC: ulcerative colitis; CD: Crohn's disease.

## Data Availability

The data used to support the findings of this study are available from the corresponding author upon request.
